# The Combination of Monochromatic LEDs and Elicitation with Stressors Enhances the Accumulation of Glucosinolates in Mustard Sprouts with Species-Dependency

**DOI:** 10.3390/plants11212961

**Published:** 2022-11-02

**Authors:** Carla Guijarro-Real, Lorena Hernández-Cánovas, Ángel Abellán-Victorio, Oumaima Ben-Romdhane, Diego A. Moreno

**Affiliations:** 1 Phytochemistry and Healthy Food Laboratory, Food Science and Technology Department, Centro de Edafología y Biología Aplicada del Segura (CEBAS), CSIC, University Campus of Espinardo, 25, Espinardo, 30100 Murcia, Spain; 2 Instituto de Conservación y Mejora de la Agrodiversidad Valenciana (COMAV), Universitat Politècnica de València, 46022 Valencia, Spain; 3 Sakata Seeds Iberica S.L., Dolores de Pacheco, 30739 Murcia, Spain

**Keywords:** black mustard, elicitor, Ethiopian mustard, food, glucosinolates, LED, white mustard

## Abstract

This work studies the enhancement of glucosinolates (GSLs) in mustard sprouts as health promoters. Sprouts of *Sinapis alba*, *Brassica nigra*, and *B. carinata* were grown under broad-spectrum, monochromatic blue or red light-emitting diode (LED) lamps, irrigated with 0–100 mM sodium chloride (NaCl), and sprayed with 0–250 µM methyl jasmonate (MeJA) as elicitor. The use of LEDs did not result in increased sprout biomass in any case. The effect of the applied treatments on the GSLs depended on the species and were restricted to *Brassica* spp. The red LEDs produced an overall increase in GSLs over 500% in *B. carinata* (from 12 to 81 mg 100 g^−1^ F.W.), compared to the white broad-spectrum lights, although the highest increase in content was obtained in treated sprouts with 250 µM MeJA (104 an 105 mg 101 g^−1^ F.W., under the red and blue LEDs, respectively). The combination of blue LEDs, 100 mM NaCl, and 250 µM MeJA enhanced the levels of GLSs in *B. nigra* to the maximum (81 mg 100 g^−1^ F.W.). Overall, these results indicate that by modifying the growing conditions for a given sprout, enhancement in the accumulation of GSLs as health promoters is possible. The use of these treatments is a sustainable alternative to genetic modification when looking for bioactive-enriched foods, delivering natural plant foods rich in bioactive ingredients (e.g., glucosinolates). Nevertheless, the response to the treatments varies among species, indicating that treatments will require adjustment across sprouts. Further research continues with producing cruciferous sprouts to obtain GSL-enriched formulas for further studying the effects of their bioavailability and bioactivity on health-promotion.

## 1. Introduction

Cruciferous vegetables are already well known for their wealth of nutrients and health-promoting bioactive compounds, specially glucosinolates (GSLs), which are family-specific metabolites [1,2,3], mainly incorporated into the human diet through the intake of Brassicaceae foods. Upon tissue disruption, for example, by mastication, these sulphur- and nitrogen-containing compounds enzymatically hydrolyse into several reactive products, such as isothiocyanates, which are linked to biological activities such as being anti-inflammatory [4] or antitumoral [5]. The biological activities of GSL-derived products would, therefore, contribute to the link established between the consumption of cruciferous foods and a lower risk of disease [6], thus reinforcing the dietary importance of these vegetables. Furthermore, these compounds also determine the pungent and bitter flavours that are characteristic of these vegetables [7], so their abundance clearly influences their acceptance.

The biosynthesis of individual GSLs and their accumulation is genetically determined by the species, cultivar, and accession [8,9,10], but also influenced by the growing conditions, as well as the ontogeny as pre-harvest factors. In this sense, several authors have corroborated the higher quality of edible Brassicaceae sprouts and young shoots compared to mature crops, especially in terms of GSLs accumulation [11,12,13]. Consequently, their use in diets and gastronomy has been popularized due to their visual attractiveness and intense flavour as a decorative complement, but also in response to their nutritional quality as a rich source of glucosinolates. Among them, allyl isothiocyanate, which is derived from the GSL sinigrin, has proved to show a positive effect against certain cancers [14] and also has biocide potential [15].

In the 2000s, it was demonstrated that the use of growth chambers provides uniform conditions for growing sprouts under a controlled environment [16,17], and the accumulation of GSLs in sprouts could be modified with light exposure [18,19]. The introduction of light-emitting diode (LED) technology and substitution of classical fluorescent light in controlled growth conditions has been extended over the last decades but intensified in the last few years, especially for indoor food production, providing higher energy efficiency and improved light quality [20]. Furthermore, LED technology allows selecting differential wavelengths and intensities that can exert a response in plant development. Within the visible light spectrum, the blue (400–500 nm) and red (600–700 nm) lights are perceived by specific photoreceptors and in the chloroplasts via chlorophylls, and exert key roles in the photosynthetic system. Consequently, these spectral regions are essential for plant growth and development, so the manipulation of the light in favour of these specific spectra has potential use in food production indoors. In fact, works measuring such an interaction with crop production and the synthesis and accumulation of primary and secondary metabolites have proliferated in the recent years. As an example, Alrifai et al. [21] reviewed the recent achievements in the modulatory effects of LED lights on microgreen production and quality.

In addition to light exposure, the application of elicitors can be also considered for enhancing the accumulation of metabolites such as GSLs. Methyl jasmonate (MeJA) is the volatile methyl ester of jasmonic acid, and an elicitor previously used in cruciferous sprouts [16] and adult plants [22]. The application of MeJA as an elicitor produced a positive increase in GSLs in broccoli sprouts and cabbage sprouts [16,17,23]. Furthermore, MeJA as a cellular regulator is involved in plant defence under biotic and abiotic stresses [22,23], such as saline environments. Thus, in those works it was found that the elicitation with MeJA showed a positive effect under salinity and enhanced the levels of GSLs, suggesting that this treatment could be exploited for the development of enriched sprouts. However, the works analysing these effects are still scarce and mainly focused on broccoli, a crop that is moderately sensitive to salinity.

In this context, our work was aimed at studying the influence that (a) specific light spectra (red or blue lights), (b) elicitation with a high dose of MeJA, and (c) saline irrigation have on the accumulation of GSLs in three mustard species. Determining the conditions that enhance the levels of these metabolites to the maximum allows the commercial production of Brassica sprouts with different GSL profiles that are specifically enriched in these compounds.

## 2. Results and Discussion

Edible sprouts are nowadays of high popularity for their attractiveness in the domestic and elite cuisine, and even more important due to their high nutritional value, being a fresh food and source of health-promoting compounds. Besides their intrinsic high value, the application of stressors at the germination stage has been studied as a potent tool to enhance the accumulation of these metabolites to obtain fresh and naturally functional sprouts.

### 2.1. Effect of Treatments on the Biomass Production

**Figure 1:**
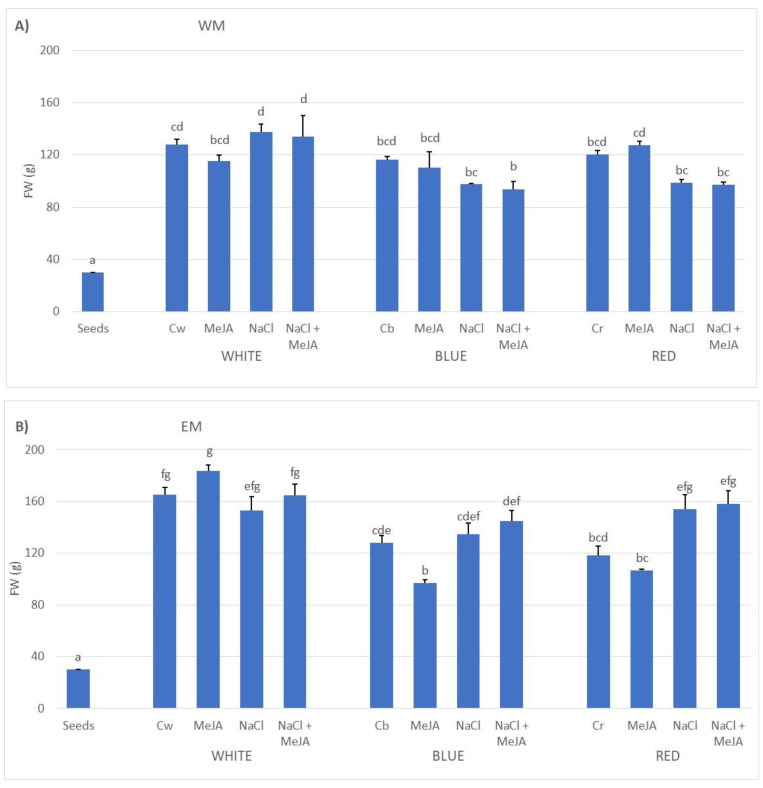

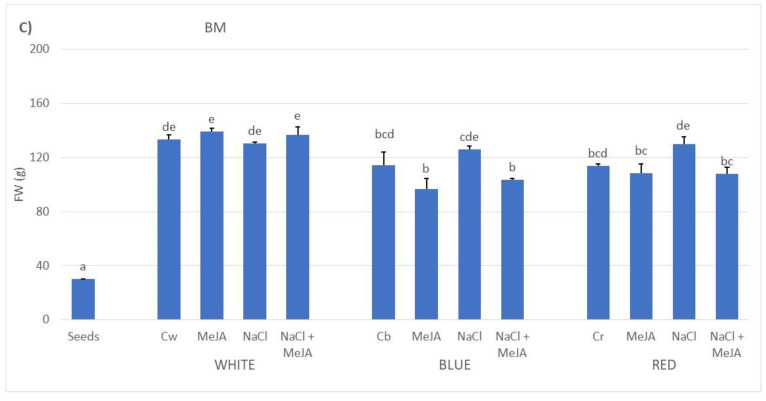
Average biomass production of the 7-day-old sprouts under three different LED lighting conditions (white or broad spectrum, blue light, and red light), with irrigation or not with a 100 mM NaCl solution, and elicitation or not with 250 µM methyl jasmonate. (**A**) White mustard. (**B**) Ethiopian mustard. (**C**) Black mustard. Cw: control, white broad-spectrum LEDs, Cb: control under blue LEDs, Cr: control under red LEDs. Different letters mean significant differences at *p* < 0.05, according to the Student–Newman–Keuls test.

In the current study, the effect of selected conditions and stressors on the sprouting and glucosinolates (GSLs) accumulation of three mustard species was evaluated. The germination of the three species led to an increase in biomass after 7 days of sprouting, on average 3.8-fold, 4.7-fold, and 4-fold the weight of the imbibed seeds for white mustard, Ethiopian mustard, and black mustard, respectively (Figure 1). The application of selected LED lighting contributed the most to the differential growth of sprouts, as the partition of the total sum of squares revealed (34–48% TSS, Table 1). Within the total spectrum, blue and red regions are specifically absorbed by the photosynthetic pigments and photoreceptors, thus influencing the photosynthesis, with many morphogenetic responses that regulate the normal plant growth and development [20]. Consequently, these regions lately have been used in a number of studies aimed to optimize conditions for growing microgreens and baby leaves, and their effect in mustard sprouts was tested in the current study. In addition to it, we also found that a second factor interacted in all cases, with lighting affecting the development of the sprouts, but whose contribution varied with the species (12.8–24.1% TSS, Table 1).

In general terms, the use of monochromatic blue or red LEDs did not improve the biomass production, and these conditions might even have led to a slight biomass reduction, as was clear for the Ethiopian mustard. Thus, the highest production in white mustard was mainly obtained for sprouts growing under broad-spectrum LEDs, with independency of the treatment applied. However, the combination of monochromatic lighting and the application of a saline stressor reduced the biomass by approximately 29% compared to the saline irrigation under experimental lights (Figure 1). Similarly, the use of monochromatic lighting also negatively affected the biomass of Ethiopian mustard sprouts, with even more significant effects. In this case, however, the saline irrigation counteracted this negative effect, especially under red lighting (Figure 1). Finally, the biomass production of black mustard was mainly affected by the differential lighting and its combination with 250 µM MeJA as elicitor (Table 1). Again, the use of monochromatic lighting reduced the total biomass of the sprouts, especially when elicitation with MeJA was also applied (an approximately 24% reduction compared to elicitation under experimental LEDs) (Figure 1).

Previous works have reported that the use of either monochromatic blue or red LEDs may affect several growth parameters in broccoli sprouts, but did not produce a biomass increase [24,25]. Neither the supplementation of warm-white light with blue or red LEDs (50 µmol m^−2^ s^−1^) was effective for increasing the biomass in baby-leaf lettuce [26]. Furthermore, even the combination of red/blue light may require supplemental broad-spectral energy of 500–600 nm (red–blue–white lighting) to increase the biomass in leafy crops, as the works of Lin et al. and Piovene et al. [27,28] suggest. However, other works found a positive effect for baby-leaf crops growing under these light regions, especially if high proportions of red lighting (>75%) were supplemented with blue LEDs at optimal ratios, instead of using monochromatic lighting [29,30]. In Ethiopian mustard microgreens (14-days old), the combination of red:blue LEDs 1:1 was enough to increase the biomass compared to monochromatic conditions [31], so these conditions could be tested in future works with 7-day-old mustard sprouts if the purpose is increasing the total biomass.

### 2.2. Profile of GSLs in the Three Mustard Species

Mustard sprouts represent a valuable condiment of intense organoleptic quality and are a rich source of GSLs with health-promoting properties. However, this foodstuff has not been deeply studied, unlike other Brassicaceae sprouts and microgreens, and the literature in terms of their nutritional composition is scarce.

In this work, the total levels of glucosinolates under control conditions (i.e., broad-spectrum lighting and no use of elicitors) were 75.31, 6.52, and 12.03 mg 100 g^−1^ FW for white, black, and Ethiopian mustard, respectively. Similar or higher levels have been determined in sprouts of white mustard [24,32,33] and black mustard [34], and in seeds and leaves of Ethiopian mustard [35,36], while Abellán et al. [37] reported total accumulation of glucosinolates in white mustard below 19 mg 100 g^−1^ FW. These differences among the published results evidence the various influencing parameters, such as the genotype, type of seeds, manufacturing conditions, and germinating conditions; indeed, even the age of the sprouts can influence the accumulation of GSLs, thus suggesting an opportunity for improvement by the proper selection and optimization of these factors.

Besides the total contents of GSLs, the individual GSLs in Brassicaceae foods are also determinant for their taste and for their expected beneficial effects on health linked to their consumption; thus, their study becomes imperative (Table 2). In the current work, the aromatic glucosinalbin (4-hydroxybenzyl-GSL) had a between 74.4% and 81.3% relative abundance in the profile of white mustard (Sinapis alba), depending on the specific growing conditions of the sprouts. In agreement, this glucosinolate typically accounts for >85% of the total profile [18,33], and was even identified as the only glucosinolate in the work of Arroyo et al. [38]. In addition, the indolic glucobrassicin (3-indolylmethyl-GSL) and 4-methoxyglucobrassicin (4-methoxy-3-indolylmethyl-GSL) accounted on average for 7.3% and 14.2% of the profile, respectively. Other minor aliphatic and aromatic glucosinolates have been reported in seeds, sprouts, and leaves of white mustard [8,32], but were not determined in our sprouts. On the other hand, the Brassica species mustards accumulated the aliphatic sinigrin instead, as described in the literature. In Ethiopian mustard, sinigrin accounted for 36.9–59.2% of the relative abundance. The indolic 4-methoxyglucobrassicin, neoglucobrassicin and 4-hydroxy-glucobrassicin were also components of this profile, with average abundances of 28.7%, 14.5%, and 8.6%, respectively. Cámara-Martos et al. [8] found that leaves of mature plants mainly accumulated sinigrin, reaching 98% of the profile, with the indolic glucosinolates being the minor components. Finally, the black mustard samples were defined by three GSLs: the aliphatic sinigrin as the main GSL, accounting for 45.6–64.1% of the profile, followed by the indolic 4-hydroxyglucobrassicin (4-hydroxy-3-indolylmethyl) and 4-methoxyglucobrassicin (11.6% and 26.3% on average, respectively). In addition, the indolic glucobrassicin and neoglucobrassicin (N-methoxy-3-indolylmethyl) only accumulated under specific treatments, and these compounds reached up to 5.3% and 21.8% under blue LED lighting.

### 2.3. Effect of LED Lighting, Saline Stressor, and MeJA Elicitor on the Accumulation of GSLs

**Figure 2:**
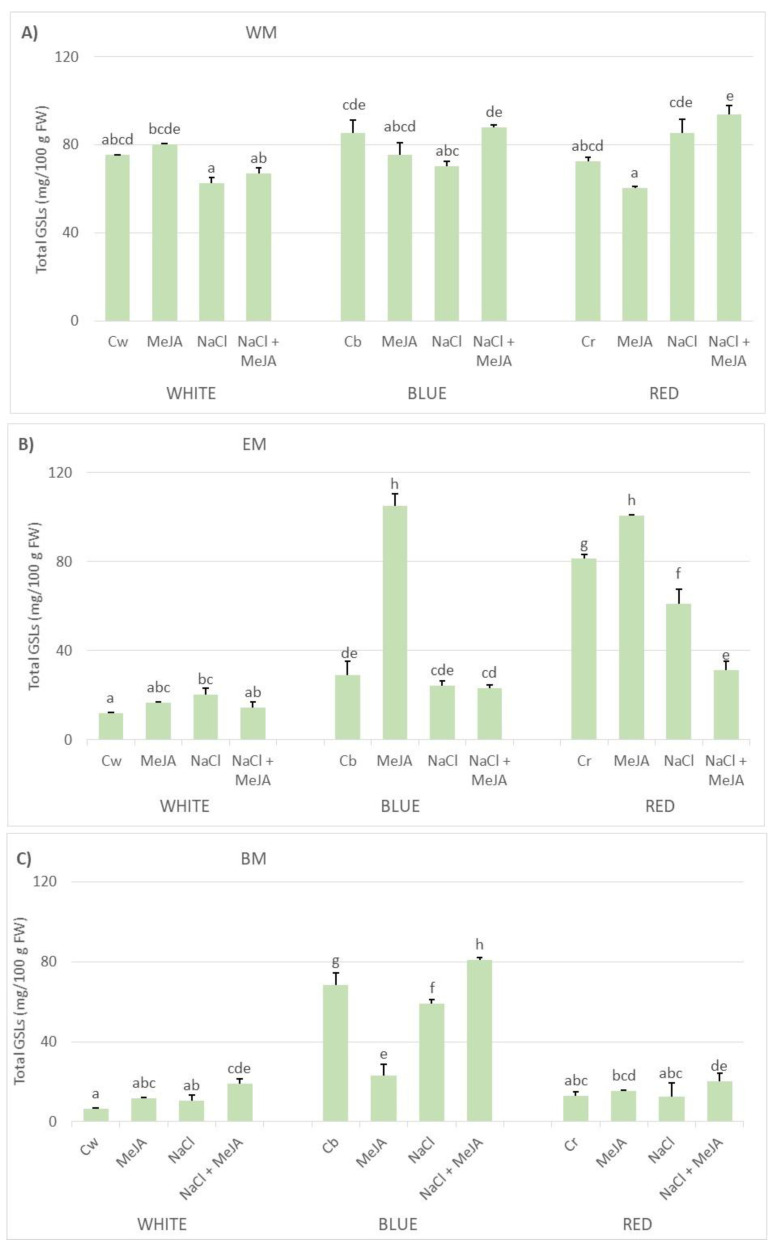
Average accumulation of glucosinolates in 7-day-old sprouts under three different LED light conditions (WHITE or broad-spectrum; blue light, BLUE; and red light, RED), with irrigation or not with a 100 mM NaCl solution, and with elicitation or not with 250 µM methyl jasmonate (MeJA). (**A**) White mustard. (**B**) Ethiopian mustard. (**C**) Black mustard. C: control, for each LED light condition (Cw, Cb, Cr). Different letters mean significant differences at *p* < 0.05, according to the Student–Newman–Keuls test.

One strategy that has proved to be successful for enriching Brassicaceae crops and sprouts in secondary metabolites is the application of exogenous elicitors, such as specific plant hormones [39], or their development under abiotic stressors, such as irrigation with saline solutions [40]. Growing these species under specific light conditions can modify the accumulation of metabolites as well [25]. In the current study, the effect that (i) differential lighting, (ii) saline irrigation, and (iii) the exogenous application of MeJA have on the biosynthesis and accumulation of glucosinolates in three mustard sprouts was tested. Table 3 summarizes the partition of the total sums of squares (TSS) into the factors studied and their interactions, and it was found that the effect was dependent on the species studied. Thus, depending on the treatments, the total glucosinolates ranged between 62.47 and 93.67 mg 100 g^−1^ for white mustard, between 12.03 and 104.88 mg 100 g^−1^ for Ethiopian mustard, and between 6.52 and 80.80 mg 100 g^−1^ for black mustard (Figure 2).

Among the three species, white mustard was found to be the most stable across treatments. As showed in Table 3, it was not the main factors but their interaction that had the highest effects, mainly the LED lighting × saline irrigation (45.3% TSS) followed by the saline irrigation × elicitation interaction (12.5% TSS), with also a significant effect due to the interaction of the three factors (7.1% TSS). While the differential lighting did not produce significant changes in the levels of glucosinolates, the combined use of saline irrigation and MeJA elicitation under blue and red LEDs increased the total levels by 31–40% compared to the application of the same treatment under broad-spectrum lighting, especially under red LEDs, which produced the highest levels (93.7 mg 100 g^−1^ F.W.). Nevertheless, it only represented a slight enhancement compared to sprouts germinated under the broad-spectrum lighting and non-saline conditions (Figure 2).

Regarding individual compounds, neither the aromatic glucosinalbin nor the indolic glucosinolates were significantly affected by the lighting, saline irrigation, or the use of MeJA as plant hormone (Table 4).

In previous works, Bennett et al. [41] did not find evidence that treating seedlings (either under dark or light conditions) or plants with 100 µM MeJA stimulated the levels of glucosinalbin due to the specific biosynthesis pathway of this compound in white mustard. In agreement, Hernández-Cánovas et al. [24] reported a slight but non-significant increase in glucosinolates when sprouts were elicited with 250 µM MeJA under broad-spectrum lighting. In our study, we tested whether combining this elicitor with an additional saline stress and specific light regions was able to promote such accumulation due to combined effects. However, no positive results were found either. Thus, our results suggest that future works attempting to enhance the profile of glucosinolates in white mustard should consider other elicitors and combinations.

Contrarily, the use of differential lighting and the application of saline stressors or plant hormones had a more marked response on the other two mustards tested, which was also species dependent. Specifically, the accumulation of glucosinolates in Ethiopian mustard developed under broad-spectrum lighting was significantly lower than white mustard, below 20.5 mg 100 g^−1^ FW, even when the elicitors were applied (Figure 2). However, the use of monochromatic LEDs had a general positive effect, this factor being a great contributor to the TSS (42.5%) (Table 3). While the application of blue LEDs alone produced a slight increase, only over 2-fold, it was the red LEDs that increased the levels by more than 500% (Figure 2). This was the result of an overall higher accumulation of indolic glucosinolates (376.6% increase) but mainly the aliphatic sinigrin (855.1% increase) (Table 5). Furthermore, spraying with 250 µM MeJA for 4 days produced the highest enhancement under either blue or red LEDs (approximately 103 mg g^−1^), while the use of saline irrigation did not have any effect (blue LEDs) or was even negative (red LEDs) for the content in glucosinolates. Furthermore, the combined use of 100 mM NaCl and 250 µM MeJA was especially negative under red LEDs (Figure 2, Table 5).

Finally, black mustard did not respond to the monochromatic red lighting, while the use of blue LEDs displayed the highest positive effect on the total accumulation of glucosinolates, more than 10-fold compared to the broad-spectrum lighting. This factor was, indeed, the main contributor to the TSS (72.0%) for the species (Figure 2, Table 6). Again, such an increase corresponded to an overall enhancement of the individual glucosinolates, mainly sinigrin (39.0 mg 100 g^−1^). Among the indolic glucosinolates, the accumulation of glucobrassicin and neoglucobrassicin, which were not identified under the broad-spectrum lighting, is relevant, especially for the latter, which reached 15 mg 100 g^−1^ due to the blue LEDs stimulus (Table 6). Furthermore, unlike the Ethiopian mustard, the combination of saline irrigation and MeJA elicitation under monochromatic blue LEDs produced an additional 18% increase in the total levels (80.8 mg 100 g^−1^ FW) due to the enhancement of sinigrin (25%) and 4-hydroxyglucobrassicin (32%), even if these elicitors alone did not seem adequate (Figure 2).

The influence of narrow-band lighting on the phytochemical composition of mustards (Brassica spp.) has been little studied compared to other sprouts. For instance, Park et al. [42] found that the use of differential lighting in 1-week-old B. juncea sprouts was not effective, mainly due to the low effect on sinigrin, as it was the main compound, despite the fact that some indolic glucosinolates increased under red LEDs. Contrarily, in the work of Maina et al. [31], both blue and red LEDs enhanced the accumulation of sinigrin in Ethiopian mustard, where especially the red LEDs were also effective in increasing the accumulation of 4-hydroxyglucobrassicin, 4-methoxyglucobrassicin, and neoglucobrassicin (while decreasing the levels of glucobrassicin), in concordance with our results. However, we also found that the use of blue LEDs produced better results than red LEDs for black mustard sprouts, as previously determined, for example, in broccoli sprouts [25]. A significant positive effect of blue LEDs on the biosynthesis of aliphatic glucosinolates in broccoli sprouts has been related with the overexpression of the CYP79F1 and CYP83A1 genes that are involved in their biosynthesis pathway [43], which may be extended to other species. In other studies with *Brassicaceae*, however, the positive effect of using monochromatic lighting could not be contrasted [44]. The controversial effects of blue or red LEDs on the accumulation of glucosinolates suggests a species dependency. Thus, our results indicate that it is not feasible to generalize in the application of specific light treatments for enhancing the phytochemical composition of sprouts, but it should be tested for the crop considered instead. Furthermore, future studies determining whether such differences correspond to a differential upregulation of the genes involved in the synthesis pathways could help in the understanding of this specificity.

As studied for white mustard, the use of differential lighting was combined with the application of stressors to determine the most adequate conditions in the Brassica mustards. As an elicitor, MeJA has been proved to enhance the nutritional quality of sprouts, here focused on the case of glucosinolates. In fact, Baenas et al. [16] found that the exogenous application of 250 µM MeJA to broccoli sprouts enhanced the accumulation of specific aliphatic glucosinolates by up to 80%, and more importantly, the levels of indolic glucosinolates, especially neoglucobrassicin. The work of Sun et al. [39] determined that one single application of 100 µM MeJA in 30-day-old Chinese kale was able to induce the biosynthesis of the indolic glucobrassicin and neoglucobrassicin. In concordance, we found significant increases in neoglucobrassicin (563%) and 4-hydroxyglucobrassicin (689%) in Ethiopian mustard, but also an increase in the aliphatic sinigrin (340%), which was not clear in previous studies. On the other hand, the application of MeJA did not enhance the levels of GSLs in black mustard. Thus, our work suggests some type of specificity in the use of MeJA as an elicitor.

Regarding the use of a saline environment, we found that the accumulation of GSLs was negatively affected when the sprouts were irrigated with 150 mM NaCl. Similarly, the use of this dose exerted a negative effect on the accumulation of both aliphatic and indolic GSLs in broccoli sprouts [23], while no significant effects were determined in red and white cabbage despite the fact that some decreases in individual GSLs were determined with variety-dependency [17]. However, lower doses were found to enhance the levels in mature broccoli plants, which could be related to their involvement in osmotic adjustment [40]. Thus, here it may be interesting to consider the application of moderate salinity. Finally, we found that the combination of a saline stress with the elicitation with MeJA can be a potential treatment for enriching Brassica sprouts in GSLs, as the results in white and black mustard showed but not in the Ethiopian mustard. Again, this result supports the need of studying the sprouts specifically for their set purpose, and not in general, as GSLs show high specificity to certain conditions.

## 3. Materials and Methods

### 3.1. Plant Material and Experimental Conditions

Three species of mustard seeds for sprouting—certified untreated—were provided by Intersemillas S.A. (Loriguilla, Valencia, Spain): white mustard (Sinapis alba L.), black mustard (Brassica nigra (L.) W.D.J.Koch), and Ethiopian mustard (Brassica carinata A. Braun). The germination conditions were as described in Hernández-Cánovas et al. [22]. Briefly, seeds were sanitated for 1 h by immersion in 5 g L^−1^ sodium hypochlorite, and then embedded for 24 h in 5 g L^−1^ sodium hypochlorite under aeration to promote the germination. For the study, thirty grams of embedded seeds were sown in trays (30 cm × 19 cm) with inert media (GrowFelt White Pads, Anglo Recycling Technology, Ltd., Lancashire, U.K.), pre-hydrated with either 5 g L^−1^ sodium hypochlorite or 100 mM NaCl (NaCl, Sigma Aldrich), in 5 g L^−1^ sodium hypochlorite (Day 0), and placed for 48 h in darkness and high relative humidity. Three-day-old sprouts were grown in a growth chamber under a 16 h/8 h photoperiod regime, 24/18 °C, 60/80% RH, and exposed to the following treatments combining three factors: differential light, use of saline irrigation as stressor, and use of MeJA as foliar elicitor.

- Light factor. Three conditions for the development of sprouts were compared (Table 7): broad-spectrum LEDs, blue LEDs, and red LEDs. Broad-spectrum lighting (BS) was provided with the model Protect BioLED 100 W (SysLed Spain, S.L.) covering the spectrum 400–700 nm. The system was compared to the selective monochromatic blue (430–520 nm) or red (580–660 nm) experimental LED light lamps provided by Inbautek S.L. (Murcia, Spain).
- Saline stressor. Trays were irrigated daily from Day 3 with the non-saline solution (5 g L^−1^ sodium hypochlorite) (0 mM NaCl) or with 100 mM NaCl in 5 g L^−1^ sodium hypochlorite (100 mM NaCl) to maintain the humidity of the inert media.
- Foliar elicitation. From Day 3, sprouts were sprayed daily with 0 or 250 µM methyl jasmonate (MeJA, Sigma-Aldrich, Análisis Vínicos SL, Tomelloso, Spain) in 0.1% ethanol (96% *v*/*v*). As a control, non-elicited trays were sprayed daily with 0.1% ethanol. A combination of elicitation and saline stress treatment was also done. As a result, 12 treatments were applied:
- Control (non-saline irrigation and 0 µM MeJA);
- Elicitation with 250 µM MeJA;
- Saline irrigation with 100 mM NaCl;
- Combined elicitation + saline irrigation (250 µM MeJA + 100 mM NaCl), for each light system.

Sprouts of 7 days old were then harvested, weighed, and freeze-dried, and the difference between the fresh and freeze-dried weight was used to determine the percentage humidity. Three independent replicates per treatment were obtained following a randomized design under each lighting.

### 3.2. Extraction and Determination of Glucosinolates (GSLs)

Glucosinolates (GSLs) were extracted from 100 mg of freeze-dried powder and analysed according to standardized and validated method for intact glucosinolates [34], as detailed in Baenas et al. [16], and available elsewhere [4,17,40]. Briefly, GSLs were extracted with 1 mL methanol 70% (*v*/*v*) for 30 min at 70 °C, with intermediate vortex shaking every 10 min. The cool extracts were centrifuged (10,000 rpm, 15 min) and the supernatants filtered through a 0.22 µm PVDF syringe 13 mmØ filters (Analisis Vínicos, Tomelloso, Spain). The identification was performed in a HPLC-PAD-ESI-MSn using the model HPLC1200 (Agilent Technologies, Waldbronn, Germany) coupled to a mass detector Bruker UltraHCT (Bruker, Bremen, Germany). The ionisation conditions were adjusted at 350 °C and 4 kV for capillary temperature and voltage, respectively. The nebulizer pressure and flow rate of the nitrogen were 65.0 psi and 11 L min^−1^, respectively. The full scan mass covered the range from *m*/*z* 100 up to m /z 1500. Collision-induced fragmentation experiments were performed in the ion trap using helium as the collision gas, with voltage ramping cycles from 0.3 up to 2 V. Mass spectrometry data were acquired in the negative ionisation mode. MSn was carried out in the automatic mode on the more abundant fragment ion in MS(n-1). Chromatograms were set at 227 nm. The quantification of GSLs was performed with a HPLC-DAD (HPLC1100 (Agilent Technologies, Waldbronn, Germany), using external standards of sinigrin and glucobrassicin (Phytoplan, Diehm und Neuberger GmbH, Heidelberg, Germany), for aliphatic and indolic glucosinolates, respectively. The compounds were separated on a Luna C18 column (250 mm × 4.6 mm, 5 μm particle size; Phenomenex, Macclesfield, UK). The mobile phase was a mixture of (A) water/trifluoroacetic acid (99.9:0.1 *v*/*v*) and (B) acetonitrile/trifluoroacetic acid (99.9:0.1 *v*/*v*). The flow rate was 0.8 mL min^−1^ in a linear gradient, starting with 1% B for 5 min to reach 17% B at 15 min, which was maintained for 2 min, then 25% B at 22 min, 35% B at 30 min, 50% B at 35 min, and 99% B at 40 min. The individual and total content of GSLs were determined and the results expressed in mg per 100 g of fresh weight (F.W.).

### 3.3. Statistical Analysis

Considering the intrinsic differences among the mustard species in terms of accumulation of individual ant total glucosinolates, independent analyses for each variety were performed. Data were subjected to a multivariate analysis of variance (ANOVA) to test the effects of the LED lighting (L, with three levels: broad-spectrum light, monochromatic blue, and monochromatic red), saline irrigation (S, with two levels: non-saline or 100 mM NaCl), foliar elicitation (E, with two levels: no elicitation or 250 µM MeJA), and their correspondent interactions. The average levels for biomass and individual and total glucosinolates were calculated from the three replicates, and the Student–Newman–Keuls multiple range test was used to determine the significance of the differences among treatments (*p* < 0.05). The statistical analysis was run in Statgraphics Centurion 18 software.

## 4. Conclusions

Brassica sprouts are rich in GSLs, with some of them being of potential interest as flavour enhancers and due to their nutritional quality. In this work, we studied the effect that different elicitors can have on the accumulation of GSLs in mustard sprouts, with the aim of developing enriched products of high nutritional quality.

Ethiopian mustard responded to the use of monochromatic red LEDs during development by increasing the total levels over 500%, with a significant increase of the aliphatic sinigrin. Furthermore, by combining this effect with the elicitation of the sprouts with MeJA produced the most significant increase in the individual GSLs. However, the addition of a saline stress had a negative effect, which may be the result of an excess of stress that compromise the development of the sprouts. Contrarily, exposure to the monochromatic blue LEDs produced the best increase in the case of black mustard, with a remarkable increase in the levels of sinigrin and also promoting the accumulation of the indolic glucobrassicin and neoglucobrassicin. An additional increase also was determined when sprouts were submitted to saline stress and elicitation with MeJA, suggesting that these conditions should be established for growing enriched black mustard sprouts. Finally, the application of these treatments in white mustard did not produce a significant response in the accumulation of GSLs, indicating that other stressors should be considered in the case of this species for obtaining enriched sprouts.

## Figures and Tables

**Table 1:** Total sum of squares (TSS, in percentage, %) for the effects of LED lighting (L: white, blue, or red LED lights), irrigation with 0 or 100 mM NaCl (S), and elicitation with spraying 0 or 250 µM MeJA (E), the interactions among them, and the residuals (R), in the biomass production of white mustard, Ethiopian mustard, and black mustard.

Species	Factor							
	L	S	E	L × S	L × E	S × E	L × S × E	R
White mustard	34.0 ***	7.9 *	1.0 ^ns^	24.1 ***	1.7 ^ns^	0.0 ^ns^	1.1 ^ns^	30.2
Ethiopian mustard	41.8 ***	11.5 ***	0.0 ^ns^	21.0 ***	4.1 ^ns^	2.4 ^ns^	3.3 ^ns^	15.8
Black mustard	48.1 ***	2.3 ^ns^	8.6 **	2.9 ^ns^	12.8 **	1.3 ^ns^	1.3 ^ns^	22.7

^ns^, *, **, and ***: not significant, or significant at *p* < 0.05, <0.01, and <0.001, respectively.

**Table 2:** Intact glucosinolates (GSLs) detected and quantified in the mustard samples.

RT (min.)	Compound	Parental Ion (ESI-) M-	GLS Semi-Systematic Name	Class	*S. alba* White Mustard	*B. nigra* Black Mustard	*B. carinata* Ethiopian Mustard
6.3	Sinigrin (SIN)	358	2-propenyl-GSL	Aliphatic		√	√
11.4	Glucosinalbin (GSB)	424	4-hydroxybenzyl-GSL	Aromatic	√		
16.3	4-Hydroxiglucobrassicin (4-HGB)	463	4-hydroxy-3-indolylmethyl-GSL	Indolic		√	√
20.6	Glucobrassicin (GB)	447	3-indolylmethyl-GSL	Indolic	√	√	
25.5	4-Methoxyglucobrassicin (4-MGB)	477	4-methoxy-3-indolylmethyl-GSL	Indolic	√	√	√
27.3	Neoglucobrassicin (NGB)	477	N-methoxy-3-indolylmethyl-GSL	Indolic		√	√

**Table 3:** Total sum of squares (in percentage, %) for the effects of LED lighting (L: experimental, blue, or red light), irrigation with 0 or 100 mM NaCl (S), and elicitation with 0 or 250 µM MeJA (E), the interactions among them, and the residuals (R), in the total accumulation of glucosinolates in white mustard, black mustard, and Ethiopian mustard.

Species	Factor							
	L	S	E	L × S	L × E	S × E	L × S × E	R
White mustard	10.7 **	1.6 ^ns^	1.0 ^ns^	45.3 ***	1.6 ^ns^	12.5 ***	7.1 *	20.1
Ethiopian mustard	42.5 ***	18.4 ***	2.5 ***	11.4 ***	8.4 ***	11.9 ***	4.2 ***	0.7
Black mustard	72.0 ***	4.7 ***	0.0 ^ns^	3.8 ***	2.9 ***	6.5 ***	9.1 ***	1.0

^ns^, *, **, and ***: not significant, or significant at *p* < 0.05, <0.01, and <0.001, respectively.

**Table 4:** Individual glucosinolates in white mustard sprouts accumulated under three LED lighting regimes (broad-spectrum, blue, or red LEDs) and the use of saline irrigation (100 mM NaCl) and spray elicitation with 250 µM MeJA.

Treatment	Glucosinolates (mg 100 g^−1^ FW)				
	GSB	GB	4-MGB	Aromatic	Indolic
*White*					
Cw	58.87 ^abc^	6.38 ^abc^	10.06 ^ab^	58.87 ^abc^	16.44 ^abc^
250 µM MeJA	64.73 ^bcd^	7.58 ^c^	8.04 ^a^	64.73 ^bcd^	15.62 ^abc^
100 mM NaCl	50.29 ^a^	5.57 ^abc^	6.61 ^a^	50.29 ^a^	12.18 ^a^
NaCl + MeJA	52.59 ^ab^	6.95 ^bc^	7.39 ^a^	52.59 ^ab^	14.35 ^abc^
*Blue LEDs*					
Cb	64.64 ^bcd^	7.17 ^bc^	13.59 ^bc^	64.64 ^bcd^	20.77 ^c^
250 µM MeJA	59.40 ^abc^	5.34 ^abc^	10.63 ^ab^	59.40 ^abc^	15.96 ^abc^
100 mM NaCl	52.36 ^ab^	3.80 ^ab^	14.24 ^bc^	52.36 ^ab^	18.05 ^abc^
NaCl + MeJA	67.51 ^cd^	6.19 ^abc^	14.11 ^bc^	67.51 ^cd^	20.30 ^c^
*Red LEDs*					
Cr	58.93 ^abc^	3.17 ^a^	10.39 ^ab^	58.93 ^abc^	13.56 ^ab^
250 µM MeJA	47.01 ^a^	5.52 ^abc^	7.96 ^a^	47.01 ^a^	13.48 ^ab^
100 mM NaCl	68.51 ^cd^	3.83 ^ab^	12.86 ^bc^	68.51 ^cd^	16.69 ^abc^
NaCl + MeJA	74.17 ^d^	4.27 ^abc^	15.23 ^c^	74.17 ^d^	19.50 ^bc^

Different letters for individual glucosinolates (GSLs) indicate significant differences among treatments according to the Student–Newman–Keuls test (*p* < 0.05). Control for each LED light condition (Cw, Cb, and Cr). For the GSLs abbreviations refer to Table 2 (GSB, glucosinolabin; GB, glucobrassicin; 4-MBG, methoxyglucobrassicin).

**Table 5:** Individual glucosinolates in Ethiopian mustard sprouts accumulated under three LED lighting regimes (broad-spectrum, blue, or red LEDs) and the use of saline irrigation (100 mM NaCl) and spray elicitation with 250 µM MeJA.

Treatment	Glucosinolates (mg 100 g^−1^ FW)					
	SIN	4-HGB	4-MGB	NGB	Aliphatic	Indolic
*White*						
Cw	5.05 ^a^	1.27 ^a^	5.70 ^a^	-	5.05 ^a^	6.98 ^a^
250 µM MeJA	8.53 ^ab^	1.28 ^a^	4.97 ^a^	1.99 ^a^	8.53 ^ab^	8.24 ^a^
100 mM NaCl	9.67 ^ab^	1.46 ^a^	6.08 ^a^	3.26 ^ab^	9.67 ^ab^	10.80 ^a^
NaCl + MeJA	6.12 ^a^	1.03 ^a^	5.25 ^a^	2.15 ^a^	6.12 ^a^	8.43 ^a^
*Blue LEDs*						
Cb	13.18 ^bc^	1.77 ^a^	11.32 ^cd^	2.83 ^ab^	13.18 ^bc^	15.92 ^b^
250 µM MeJA	58.02 ^f^	13.94 ^e^	14.19 ^e^	18.73 ^f^	58.02 ^f^	46.87 ^f^
100 mM NaCl	8.94 ^ab^	2.10 ^a^	10.48 ^c^	2.72 ^ab^	8.94 ^ab^	15.30 ^b^
NaCl + MeJA	8.46 ^ab^	0.98 ^a^	10.02 ^c^	3.85 ^b^	8.46 ^ab^	14.85 ^b^
*Red LEDs*						
Cr	48.20 ^e^	8.77 ^c^	10.28 ^c^	14.23 ^e^	48.20 ^e^	33.27 ^d^
250 µM MeJA	56.95 ^f^	9.96 ^d^	12.58 ^d^	21.10 ^g^	56.95 ^f^	43.65 ^e^
100 mM NaCl	34.52 ^d^	7.17 ^b^	8.16 ^b^	11.15 ^d^	34.52 ^d^	26.49 ^c^
NaCl + MeJA	15.11 ^c^	1.78 ^a^	7.67 ^b^	6.53 ^c^	15.11 ^c^	15.99 ^b^

Different letters for individual GSLs indicate significant differences among treatments according to the Student–Newman–Keuls test (*p* < 0.05). Control for each LED light condition (Cw, Cb, and Cr). For GSLs abbreviations refer to Table 2 (SIN, sinigrin; 4-HGB, hydroxyglucobrassicin; 4-MGB, methoxyglucobrassicin; NGB, neoglucobrassicin).

**Table 6:** Individual glucosinolates in black mustard sprouts accumulated under three LED lighting regimes (broad-spectrum, blue, or red LEDs) and the use of saline irrigation (100 mM NaCl) and spray elicitation with 250 µM MeJA.

Treatment	Glucosinolates (mg 100 g^−1^ FW)						
	SIN	4-HGB	GB	4-MGB	NGB	Aliphatic	Indolic
*White*							
Cw	2.97 ^a^	1.40 ^a^	-	2.14 ^a^	-	2.97 ^a^	3.55 ^a^
250 µM MeJA	5.77 ^ab^	2.00 ^ab^	-	3.86 ^b^	-	5.77 ^ab^	5.86 ^ab^
100 mM NaCl	4.90 ^a^	1.14 ^a^	-	4.55 ^bc^	-	4.90 ^a^	5.69 ^ab^
NaCl + MeJA	12.14 ^b^	2.86 ^b^	-	3.92 ^b^	-	12.14 ^b^	6.79 ^ab^
*Blue LED*							
Cb	38.96 ^c^	4.75 ^c^	3.65 ^b^	6.21 ^c^	14.97 ^c^	38.96 ^c^	29.59 ^e^
250 µM MeJA	11.06 ^b^	2.36 ^ab^	-	7.69 ^d^	2.00 ^a^	11.06 ^b^	12.05 ^c^
100 mM NaCl	34.96 ^c^	4.36 ^c^	2.38 ^a^	5.25 ^bc^	12.19 ^b^	34.96 ^c^	24.18 ^d^
NaCl + MeJA	48.67 ^d^	6.26 ^d^	3.79 ^b^	5.24 ^bc^	16.83 ^c^	48.67 ^d^	32.12 ^f^
*Red LED*							
Cr	6.36 ^ab^	0.98 ^a^	-	4.79 ^bc^	0.74 ^a^	6.36 ^ab^	6.51 ^ab^
250 µM MeJA	7.61 ^ab^	1.14 ^a^	-	5.12 ^bc^	1.54 ^a^	7.61 ^ab^	7.80 ^b^
100 mM NaCl	7.17 ^ab^	1.51 ^a^	-	3.62 ^b^	0.40 ^a^	7.17 ^ab^	5.52 ^ab^
NaCl + MeJA	11.74 ^b^	3.08 ^b^	-	5.55 ^bc^	-	11.74 ^b^	8.63 ^b^

Different letters for individual glucosinolates indicate significant differences among treatments according to the Student–Newman–Keuls test (*p* < 0.05). Control for each LED light condition (Cw, Cb, and Cr) For the GSLs abbreviations refer to Table 2 (SIN, sinigrin; 4-HGB, hydroxyglucobrassicin; GB, glucobrassicin; 4-MGB, methoxyglucobrassicin; NGB, neoglucobrassicin).

**Table 7:** LED light treatments and their spectral and energy characteristics.

LED Treatment	Wavelength Spectra (nm)	Electric Intensity (mA)	Luminous Flux per Unit Area (lx)	PPFD
White light (control)	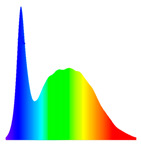 (380–780 nm)	20 mA	225	7
Red light	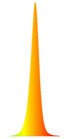 (580–660 nm)	80 mA	35	7
Blue light	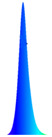 (430–520 nm)	60 mA	210	7

## Data Availability

Data are available upon request.
